# Influence of Vitamin D3 Supplementation on Infliximab Effectiveness in Chinese Patients With Crohn's Disease: A Retrospective Cohort Study

**DOI:** 10.3389/fnut.2021.739285

**Published:** 2021-10-22

**Authors:** Sheng-long Xia, Quan-jia Min, Xiao-xiao Shao, Dao-po Lin, Guo-long Ma, Hao Wu, Shu-guang Cao, Yi Jiang

**Affiliations:** Department of Gastroenterology, The Second Affiliated Hospital of Wenzhou Medical University, Wenzhou, China

**Keywords:** vitamin D, cytokines, infliximab, retrospective studies, Crohn's disease

## Abstract

**Background:** It remains uncertain whether vitamin D3 (vitD3) supplementation is beneficial for remission of Crohn's disease (CD). The influence of vitD3 supplementation on Infliximab (IFX) effectiveness was analyzed in Chinese CD patients.

**Methods:** In this retrospective cohort study, moderate-to-severe CD patients, who were bio-naïve and prescribed with IFX treatment for at least 54 weeks, were recorded from January 2014 to December 2019. VitD3 supplementation was defined as patients additionally took oral vitD3 (125 IU/d) within 3 days after the first infusion and persisted in the whole follow-up period. Disease activity was assessed using Harvey-Bradshaw Index (HBI). Serum cytokine profiles (IL-2, IL-4, IL-6, IL-10, TNF-α, and IFN-γ) were quantitatively analyzed in a subset of all patients at baseline and 54-week after intervention.

**Results:** Among 73 enrolled patients, 37 took vitD3 regularly (D3-patients), the others (non-D3-patients) did not. At 54-week, the mean 25-hydroxyvitaminD level increased in D3-patients (20.33 vs. 15.07 ng/mL, *P* < 0.001). The clinical remission rate was higher in D3-patients compared to non-D3-patients (83.8 vs. 61.6%, *P* = 0.030). The decrease of HBI from baseline to 54-week was more in D3-patients than non-D3-patients (7.41 ± 3.0 vs. 6.28 ± 2.75, *P* = 0.023). Furthermore, vitD3 supplementation was independently related to the increase of remission rate at 54-week in D3-patients (β = −1.667, *P* = 0.015). The benefit of vitD3 supplementation was significant only in patients with deficient vitD3 (all *P* < 0.05), but not in non-deficient vitD3. A total of nine patients (four non-D3-patients and five D3-patients) were selected to determine serum cytokine profiles after 54-week IFX treatment. In non-D3-patients, the decreases of TNF-α and IL-6 at 54-week were more obvious than at baseline (*P* = 0.032, 0.022, respectively). In D3-patients, however, only IL-10 increased at 54-week compared with its baseline value (*P* = 0.037).

**Conclusions:** VitD3 supplementation could improve IFX effectiveness in CD patients, especially for patients with vitD3 deficiency. This beneficial effect of vitD3 supplementation probably arose from the up-regulation of IL-10.

**Trial Registration:** NCT04606017.

## Introduction

Inflammatory bowel diseases (IBD) encompass two distinct disorders, Crohn's disease (CD) and ulcerative colitis (UC). Owing to its prolonged course and incurability, IBD has gradually evolved into a large health-care burden (1, 2). Infliximab (IFX), namely anti-tumor necrosis factor (TNF) alpha antagonist, can effectively induce and maintain the remission of CD and UC. However, it remains a clinical challenge to manage IFX loss of response, which partly due to its immunogenicity giving rise to heightened clearance and reduced drug levels (3). Current strategies to solve this include the combination of biological therapy with an immunomodulator, such as azathioprine (AZA), to achieve synergetic effect and enhance efficacy.

In addition to modulating calcium and phosphorus metabolism, vitamin D (VitD) plays a pivotal role in maintaining immune homeostasis, especially for the intestinal immune system. It has been demonstrated evidently that the intestinal epithelium depends on vitD to maintain its integrity (4). The studies with genetic knockout of vitD receptor gene (*VDR*^−/−^) in experimental models of colitis revealed that *VDR*^−/−^ mice were more prone to epithelial injury, which were typically characterized by the loss of intestinal epithelial tight junctions, breakdown in epithelial integrity, and increased bacterial penetration (5, 6). Furthermore, as a vital counterbalance to activation of adaptive immunity, vitD could suppress the proliferation of T and B cells, inhibit the expression of various pro-inflammatory cytokines (such as TNF-α), and induce regulatory T cells (Tregs) to secrete anti-inflammatory cytokines (such as IL-10) (7–9). Some of these cytokines have been proven to participate in the pathogenesis of IBD, thereby affecting the efficacy of biologic therapies (10, 11).

Several lines of evidence to date have implicated that there is a change in serum concentration of vitD in the clinical progression of CD (12, 13). Data from the northern Europe and China demonstrated that vitD deficiency were present in more than half of CD patients (14, 15). Moreover, the CD patients with vitD deficiency tended to have worse outcomes, including increased risk of hospitalization, more frequent computed tomography scans and emergency department visits, as well as increased use of steroids and biologics after diagnosis (16). Oppositely, the normalization of serum vitD may result in a lower risk of CD-related surgery when compared to those CD patients with low serum concentration of vitD (17).

Although several observational studies have implied that vitD status is related to efficacy of biological agents in IBD patients, this concept is still in dispute. For example, Reich et al. reported that CD patients initiating IFX with a low vitD level were more likely to achieve clinical remission at week 14 (18). During anti-TNF-α treatment, the patients with vitD deficiency would have earlier cessation of this therapy compared to those with normal vitD levels (19). Nevertheless, how vitD supplementation may affect IFX effectiveness in CD patients has not been investigated thoroughly. Herein we carried out this retrospective cohort study to determine (1) whether vitD supplementation in IFX-treated CD patients could raise the serum concentration of vitD, and improve the remission rate at 54-week; (2) what were the determinants for vitD supplementation in improving the response to IFX; (3) whether this supplementary treatment influenced the expression profiles of Th-cell related cytokines in CD patients under IFX treatment.

## Methods

### Study Design

This was a single-center retrospective cohort study conducted in the Second Affiliated Hospital of Wenzhou Medical University, Wenzhou city, Zhejiang province, China. Our study was performed in accordance with the guidelines of the Declaration of Helsinki and was approved by the Ethics Committee of the Hospital (Ethical Application Ref: 2021-k-37-02). This clinical trial was registered in the Clinical Trials. Gov (NCT04606017). All participants provided written informed consent.

### Patients

Patients were eligible if they received a comfirmed diagnosis of moderate-to-severe CD defined as a total score >8 on Harvey Bradshaw Index (HBI) between January 2014 to December 2019 and were bio-naïve at the time of diagnosis. The diagnosis of CD was based on accepted standard criteria including the combination of clinical symptoms, endoscopy, radiology, pathology, and surgical history (20). Extend of disease was defined using the Montreal Classification as being ileal (L1), colonic (L2), ileocolonic (L3), or upper gastrointestinal (L4), while disease behavior was stratified as non-penetrating, non-stricturing (B1), stricturing (B2), or penetrating disease (B3), with an additional modifier for perianal diseases (20). Since L4 could coexist with ileal and colonic disease (e.g., L1+L4, L2+L4, L3+L4), all such patients were categorized as both L4 and L1–L3 in this study. Patients were included if they regularly received IFX infusion for at least 54 weeks based on the recommended guideline. Patients were excluded if they (1) were <18 years older, (2) had recent supplementation of vitD3, (3) had prior or concomitant use of other biologic agent, glucocorticoid and/or immunomodulators at the time of enrollment, (4) were pregnant, (5) had cognitive/developmental disorders that affected their ability to complete the study procedures, and (6) had medical illness or therapies potentially affecting bone, nutrition or growth status, including kidney disease, seizure disorders, and liver disease. Patients who were under long-term exposure to antibiotics were also excluded.

### Data Collection

All data were retrospectively retrieved from a uniform data collection form. Data on the following variables were recorded at the time of IFX initiation (baseline) and week 54, including (1) patient characteristics: age, age of diagnosis, gender, smoking status, and body mass index (BMI); (2) disease characteristics: disease extent, disease behavior, disease activity (assessed by HBI), prior surgical history, and medications (including biologic agents, glucocorticoid, immunomodulators, and vitamin D agents); (3) biochemical parameters: hemoglobin (Hb), platelet (PLT), C-reactive protein (CRP), erythrocyte sedimentation rate (ESR), albumin (Alb), as well as serum concentration of calcium and phosphorus; (4) serum concentration of 25-hydroxyvitamin D [25(OH)D], which was a routine test item in our hospital for assessment of CD disease activity. The cut-off concentration for vitamin D deficiency was taken from the definition of the Endocrine Society Recommendations: patients with 25(OH)D below 20 ng/mL were considered deficient, while those with 25(OH)D ≥ 20 ng/mL were considered non-deficient (21).

### Intervention and Follow-Up

Treatment with IFX was administered intravenously at a dose of 5 mg/kg at 0, 2 and 6 weeks and every 8-week thereafter. The total duration of IFX treatment for each patient was at least 54 weeks.

VitD3 supplementation was defined as an oral intake of 125 IU/d vitD3 provided by Calcium Carbonate and VitD Tablets (Wyeth Pharmacy Co. Ltd). The supplementary treatment was initiated within 3 days after the first IFX infusion and persisted in the whole follow-up period.

### Outcomes

The primary outcome was the change of serum 25(OH)D concentration and disease activity at week 54. This time point choice was based on two reasons as below: (1) minimization in the influence of season on the fluctuation of vitD levels as much as possible, (2) reference to previous studies on the evaluation of IFX effectiveness (22). The secondary outcome was the metabolism of calcium and phosphorus, as well as the expression profiles of Th-cell-related cytokines at week 54.

### Cytokine Quantification

Blood samples were collected in a subset of all patients at both baseline and week 54. Serum was harvested by centrifugation (3,000 r/min for 15 min) within 1–4 h after collection of fasting blood sample, and cryopreserved at −80°C until further analysis. Serum levels of IL-2, IL-4, IL-6, IL-10, TNF-α, and IFN-γ were quantified by Cytometric Bead Array (CBA) (Cell-gene Biotech Co., LTD, JiangXi, China) and analyzed using FACS Canto II (BD Biosciences).

### Statistical Analysis

All statistical tests were performed in *SPSS* 25.0 software (SPSS, Chicago, IL, USA). For quantitative variables, data are shown as mean ± standard deviation (SD) or as median and inter-quartile range (IQR), according to the presence or absence of a normal distribution, respectively. The Shapiro-Wilk test was used to evaluate the normality. Continuous variables between D3-patients and non-D3-patients were compared using Student's *t*-test or non-parametric Wilcoxon's signed rank test as appropriate. For comparison of continuous variables between baseline and week 54, the paired *t*-test or paired Wilcoxon's signed rank test was performed according to the results of normal distributions. The chi-square test was used to compare categorical variables. Multivariable logistic regression with enter selection was modeled to determine the independent factors affecting the achievement of remission in CD patients under IFX treatment. The following variables were selected: age, gender, smoking status, disease extent, disease behavior, perianal lesions, upper GI involvement, prior surgical history, concomitant use of AZA, and vitD3 supplementation. The multivariable linear regression model (enter method) was further built to analyze the determinants for the decrease of HBI, as well as the increase of 25(OH)D in D3-patients. The variables included age, gender, smoking status, disease extent, disease behavior, perianal lesions, upper GI involvement, prior surgical history, concomitant use of AZA, BMI, Hb, PLT, CRP, ESR, Alb, baseline 25(OH)D, and HBI. Among them, smoking status, disease extent, disease behavior, perianal lesions, upper GI involvement, prior surgical history and concomitant use of AZA, were transformed as dummy variables. All variables included in the regression models were selected based on the clinical experience and the related factors reported by other researchers. The statistical significance was assumed for *P*-values < 0.05.

## Results

### Demographics Characteristics

**Figure 1:**
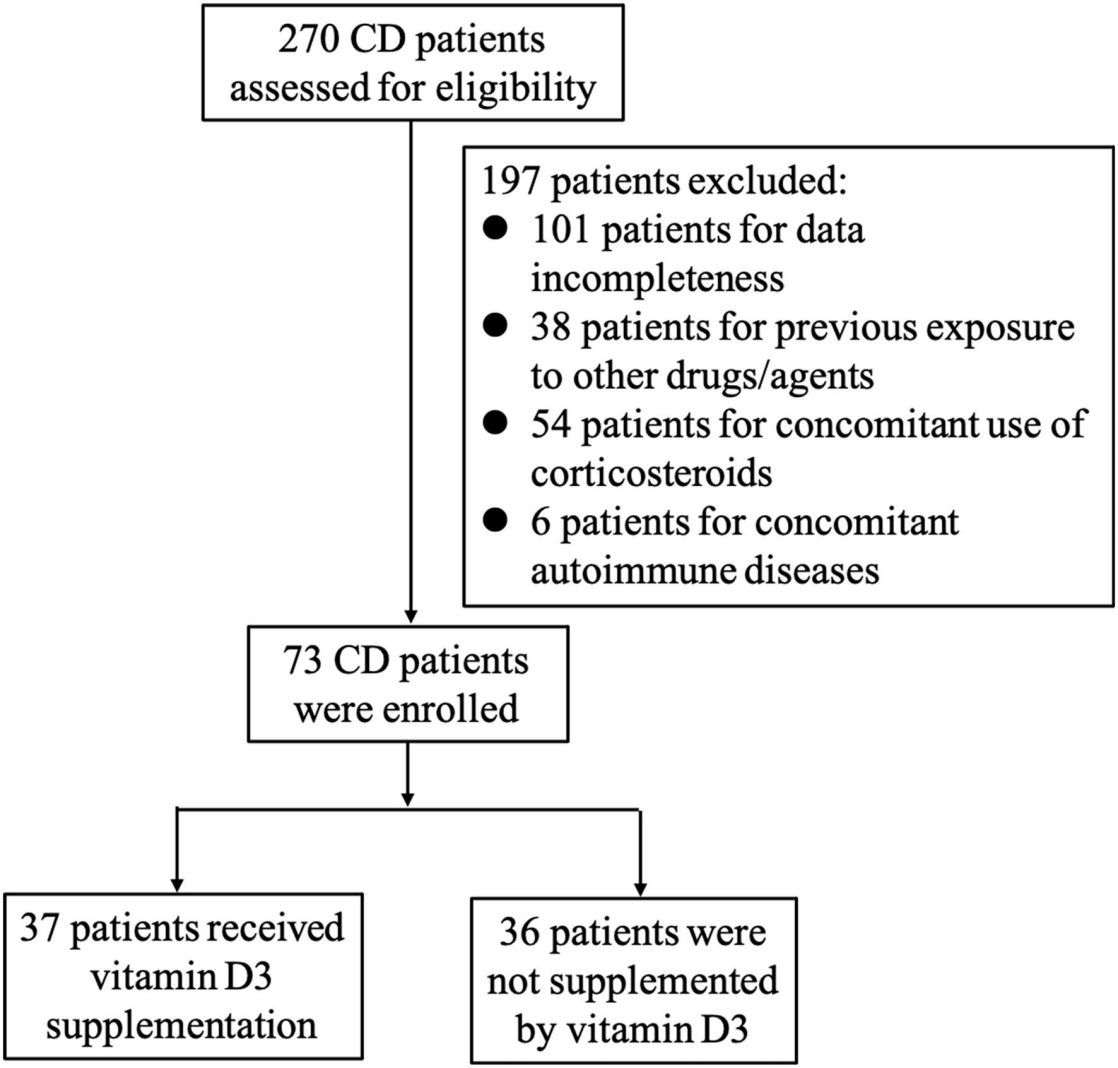
Study overview.

As presented in Figure 1, a total of 270 patients received IFX treatment for moderate-to-severe active CD in our hospital. Among them, 197 patients were excluded: 101 because of data incompleteness, 38 owing to previous exposure to other drugs/agents as aforementioned exclusive criterion, 52 due to concomitant use of corticosteroids during the administration of IFX, and six were found to have other autoimmune diseases. Finally, 73 patients were enrolled in our study.

The demographics characteristics of CD patients are summarized in Table 1. The median (IQR) age was 29 (21–37) years, and 64.4% of subjects were male. The median (IQR) age of diagnosis was 27 (20–36) years, and A2 (17–40 years older) occupied a majority of all patients (80.8%). Smoking habits presented in only 5 patients (6.8%). Most of the enrolled patients had L1 diseases and B1/B2 behaviors. Of them, 23 patients (31.5%) had perianal lesions and four patients (5.5%) had upper GI involvement. Except for two patients with severe activity, the remaining 71 patients had moderate disease activity at the time of enrollment, with a median (IQR) HBI of 10 (9–12). Seven patients (9.6%) underwent surgical intervention due to intestinal complications, and 19 patients (26.0%) started the combination therapy with AZA (1–2 mg/kg·d) at the fourth times of IFX infusion as they had the predictors related to poor prognosis, such as age <40 years old and upper GI involvement (23).

**Table 1:** Baseline characteristics.

	**Total cohort (*****n*** **=** **73)**			**Vitamin D deficient group (*****n*** **=** **50)**			**Vitamin D non-deficient group (*****n*** **=** **23)**		
	**Non-D3-patients** **(*n* = 36)**	**D3-patients** **(*n* = 37)**	***P***	**Non-D3-patients** **(*n* = 23)**	**D3-patients** **(*n* = 27)**	***P***	**Non-D3-patients** **(*n* = 13)**	**D3-patients** **(*n* = 10)**	***P***
Age, years	32.39 ± 11.84	24 (20, 36)	0.143	33.82 ± 12.76	25 (19, 34)	0.092	29.85 ± 9.97	24 (20.75, 46.25)	1
Age of diagnosis, years	29.5 (20–36.5)	23 (20–34)	0.577	31.52 ± 12.09	25 (19.5–31.5)	0.219	21 (18–35)	23 (20–44)	0.410
A1, *n* (%)	2 (5.5)	0 (0.0)		1 (4.3)	0 (0.0)		1 (7.7)	0 (0.0)	
A2, *n* (%)	28 (77.8)	31 (83.8)		17 (74.0)	24 (88.9)		11 (84.6)	7 (70.0)	
A3, *n* (%)	6 (16.7)	6 (16.2)		5 (21.7)	3 (11.1)		1 (7.7)	3 (30.0)	
Male, *n* (%)	26 (72.2)	21 (56.8)	0.168	13 (56.5)	11 (40.7)	0.845	13 (100.0%)	5 (50.0%)	**0.007**
Current smoker, *n* (%)									
Yes	4 (11.1)	1 (2.7)	0.155	3 (13.0)	1 (3.7)	0.322	1 (7.7%)	0 (0.0)	1
No	32 (88.9)	36 (97.3)		20 (87.0)	26 (96.3)		12 (92.3%)	10 (100.0)	
Disease behavior, *n* (%)									
B1	18 (50)	15 (40.5)	0.455	12 (52.5)	17 (48.1)	0.928	4 (30.8)	4 (40.0)	0.058
B2	16 (44.4)	17 (45.9)		2 (8.7)	2 (7.4)		0 (0.0)	3 (30.0)	
B3	2 (5.6)	5 (13.5)		9 (39.1)	12 (44.4)		9 (69.2)	3 (30.0)	
Disease extent, *n* (%)									
L1	21 (58.3)	15 (40.5)	0.309	13 (56.5)	10 (37)	0.383	8 (61.5)	5 (50.0)	0.700
L2	3 (8.3)	5 (13.5)		1 (4.3)	2 (7.4)		2 (15.4)	3 (30.0)	
L3	12 (33.3)	17 (45.9)		9 (39.1)	15 (55.6)		3 (23.1)	2 (20.0)	
L4*	1 (2.8)	3 (8.1)	0.317	1 (4.3)	3 (11.1)	0.380	0 (0)	0 (0)	-
Perianal lesions, *n* (%)	9 (25.0)	14 (37.8)	0.238	6 (26.1)	10 (37.0)	0.408	3 (23.1)	4 (40)	0.650
Prior surgical history, *n* (%)	4 (11.1)	3 (8.1)	0.711	3 (13.0)	2 (7.4)	0.651	1 (7.7)	1 (10.0)	1
Concomitant use of AZA, *n* (%)	6 (16.7)	13 (35.1)	0.072	4 (17.4)	10 (37)	0.123	2 (15.4)	3 (33.0)	0.618

*According to the Montreal classification, age of diagnosis in CD was defined as ≤ 16 years (A1), 17–40 years (A2), and >40 years (A3); disease extent was defined as being ileal (L1), colonic (L2), ileocolonic (L3), or upper gastrointestinal (L4); disease behavior was stratified as non-penetrating, non-stricturing (B1), stricturing (B2), or penetrating disease (B3) with a modifier for perianal involvement.*

*Since L4 could coexist with ileal and colonic disease (e.g., L1+L4, L2+L4, L3+L4), all such patients were categorized as both L4 and L1–L3 in this study.

*GI, gastrointestinal; AZA, azathioprine; HBI, Harvey-Bradshaw Index*.

**Figure 2:**
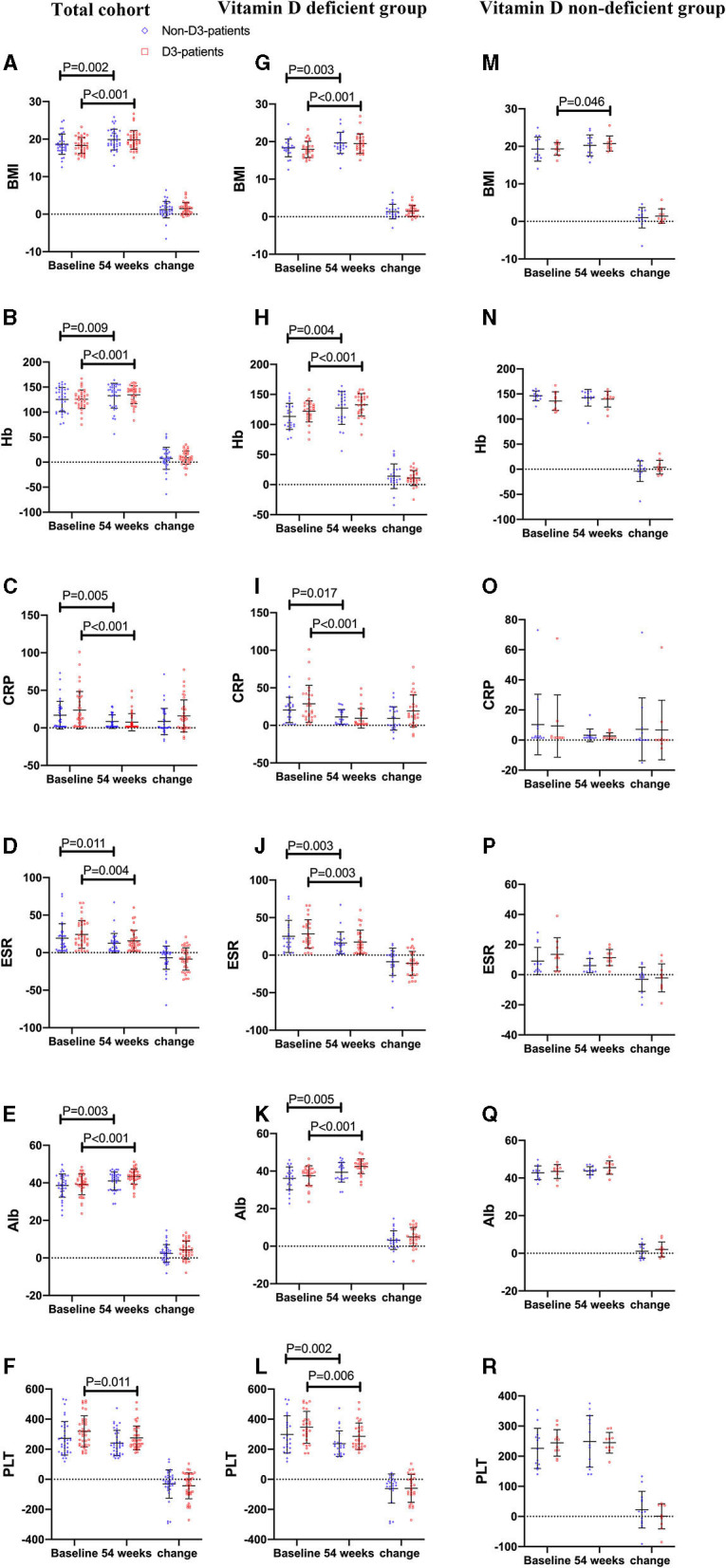
The changes of BMI and biochemical parameters between baseline and week 54 in CD patients receiving IFX therapy. **(A–F)** the changes in the total cohort. **(G–L)** the changes in the vitD deficient group. **(M–R)** the changes in the vitD non-deficient group.

Thirty-seven patients received vitD3 supplementation (D3-patients), while 36 did not (non-D3-patients). There were no significant differences in baseline characters between D3-patients and non-D3-patients (*P* > 0.05) (Table 1, Figure 2A). Serum levels of inflammatory parameters (Hb, CRP, ESR, Alb, and PLT) at baseline were also similar between the two groups (all *P* > 0.05) (Figures 2B–F).

### Influence of VitD3 Supplementation on the Change of Serum 25(OH)D

**Figure 3:**
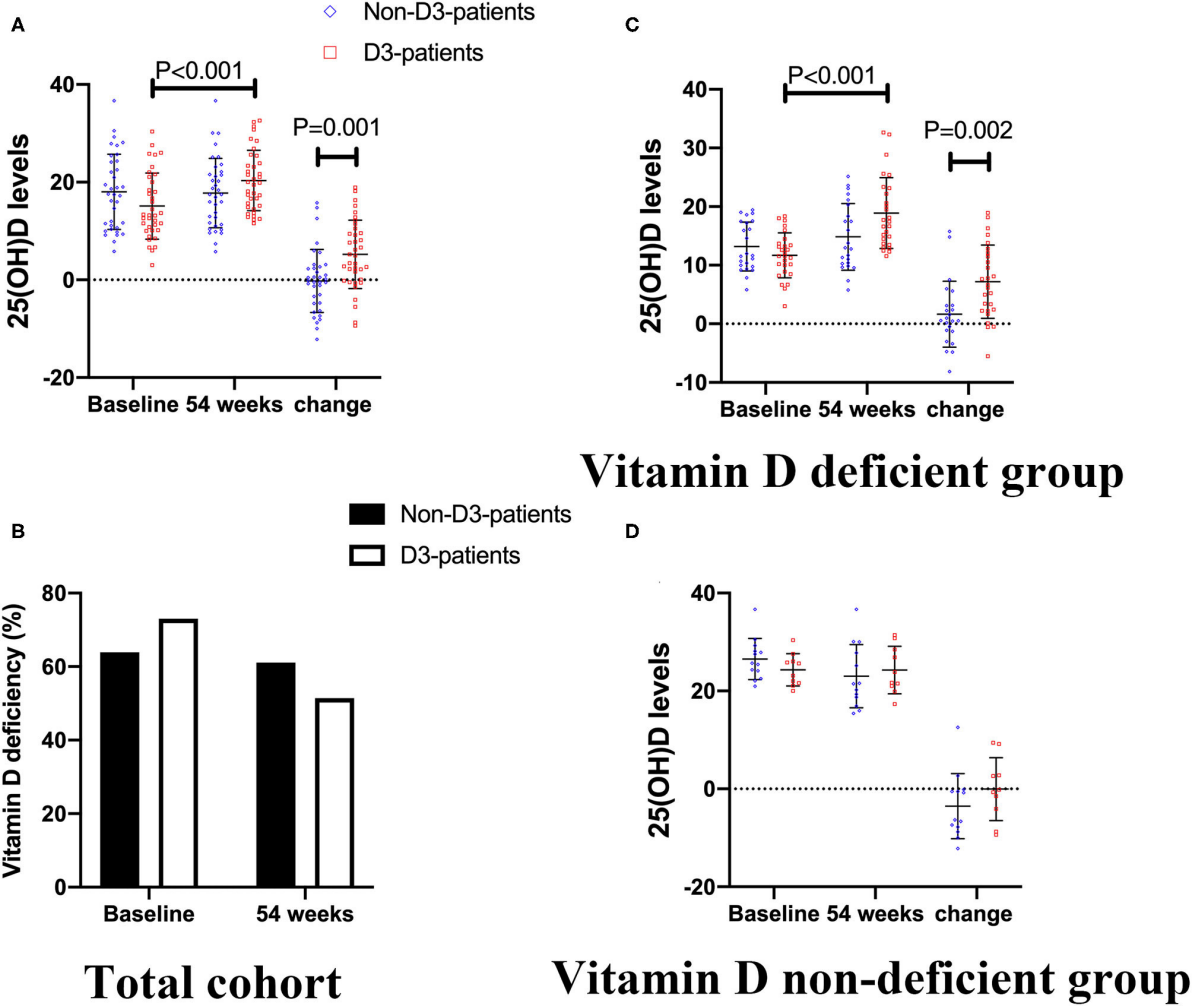
The changes of 25(OH)D levels between baseline and week 54 in CD patients receiving IFX therapy. **(A,B)** In total cohort. **(C)** In vitD deficient group. **(D)** In vitD non-deficient group.

As described in Figure 3A, the baseline level of 25(OH)D was 15.07 ± 6.70 ng/mL in D3-patients, while 18.01 ± 13.48 ng/mL in non-D3-patients (*P* = 0.086, student's *t*-test). There was a clear different change pattern of 25(OH)D between the two groups. The mean serum concentration of 25(OH)D basically stayed stable in non-D3-patients throughout the duration of the study (baseline vs. week 54: 18.01 ± 13.48 vs. 17.79 ± 7.10, *P* = 0.841, paired *t*-test). In D3-patients, the average concentration of 25(OH)D significantly raised from baseline 15.07 to 20.33 ng/mL at week 54 (*P* < 0.001, paired *t*-test). After 54-week of vitD3 supplementation, D3-patients manifested a greater increase of 25(OH)D than non-D3-patients did (5.24 ± 7.01 vs. −0.22 ± 6.44, *P* = 0.001, student's *t*-test) (Figure 3A).

Based on the above findings, the linear regression model was further established to identify the contributors for the increase of 25(OH)D in D3-patients. The independent variables including age, gender, smoking status, disease extent, disease behavior, perianal lesions, upper GI involvement, prior surgical history, concomitant use of AZA, BMI, Hb, PLT, CRP, ESR, Alb, baseline 25(OH)D, and HBI. We found that both upper GI involvement and baseline 25(OH)D concentration were negatively correlated to the increase of 25(OH)D after vitD3 supplementation (*B* = −12.797, *P* = 0.020; *B* = −1.144, *P* < 0.001, respectively) (Table 2). In other words, the patients with upper GI involvement or higher baseline 25(OH)D had a less increase of 25(OH)D after oral vitD3 supplementation.

**Table 2:** Influence factors on the increase of vitamin D levels in CD patients supplemented by vitamin D3 (Liner regression, enter) (*n* = 37).

**Covariants**	***B***	**standard error**	***t***	***P***	**95%CI for *B***
Age	0.209	0.111	1.879	0.075	−0.023–0.442
Sex^§^	2.877	3.769	0.763	0.454	−4.986–10.740
Smoking status^§^	7.454	9.306	0.801	0.433	−11.958–26.866
Perianal lesions^§^	3.268	2.526	1.294	0.211	−2.002–8.537
Upper GI involvement^§^	−12.797	5.042	−2.538	**0.020**	−23.315 −2.280
Concomitant use of AZA^§^	−0.505	2.155	−0.234	0.817	−5.001–3.991
Prior surgical history^§^	2.994	3.549	0.844	0.409	−4.408–10.397
Disease extent^§^	−0.317	1.476	−0.215	0.832	−3.396–2.761
Disease behavior^§^	0.307	1.555	0.197	0.846	−2.937–3.550
BMI	0.038	0.703	0.054	0.958	−1.428–1.504
Hb	−0.114	0.136	−0.835	0.414	−0.398–0.170
PLT	−0.047	0.024	−1.999	0.059	−0.097–0.002
CRP	0.040	0.080	0.493	0.627	−0.128–0.207
ESR	0.033	0.106	0.316	0.756	−0.188–0.255
Albumin	0.502	0.322	1.558	0.135	−0.170–1.174
Baseline levels of Vitamin D	−1.144	0.235	−4.874	** <0.001**	−1.634 −0.655

§ *Transformed as dummy variable.*

*GI, gastrointestinal; AZA, azathioprine; BMI, body mass index; Hb, hemoglobin; PLT, platelet; CRP, C-reactive protein; ESR, erythrocyte sedimentation rate*.

In addition, there was 73.0% (27/37) patients was deficient in vitD in D3-patients, and 63.9% (23/36) patients in non-D3-patients at baseline (*P* = 0.404). After 54-week of oral administration of vitD3, the frequency of vitD deficiency was reduced from 73.0 to 51.4% in D3-patients (*P* = 0.055), while from 63.9 to 61.1% in non-D3-patients (*P* = 0.808). Either in D3-patients or non-D3-patients, however, no statistical difference was found in terms of the distribution of vitD deficiency between baseline and 54-week (all *P* > 0.05) (Figure 3B).

### Influence of VitD Supplementation on Disease Activity

**Figure 4:**
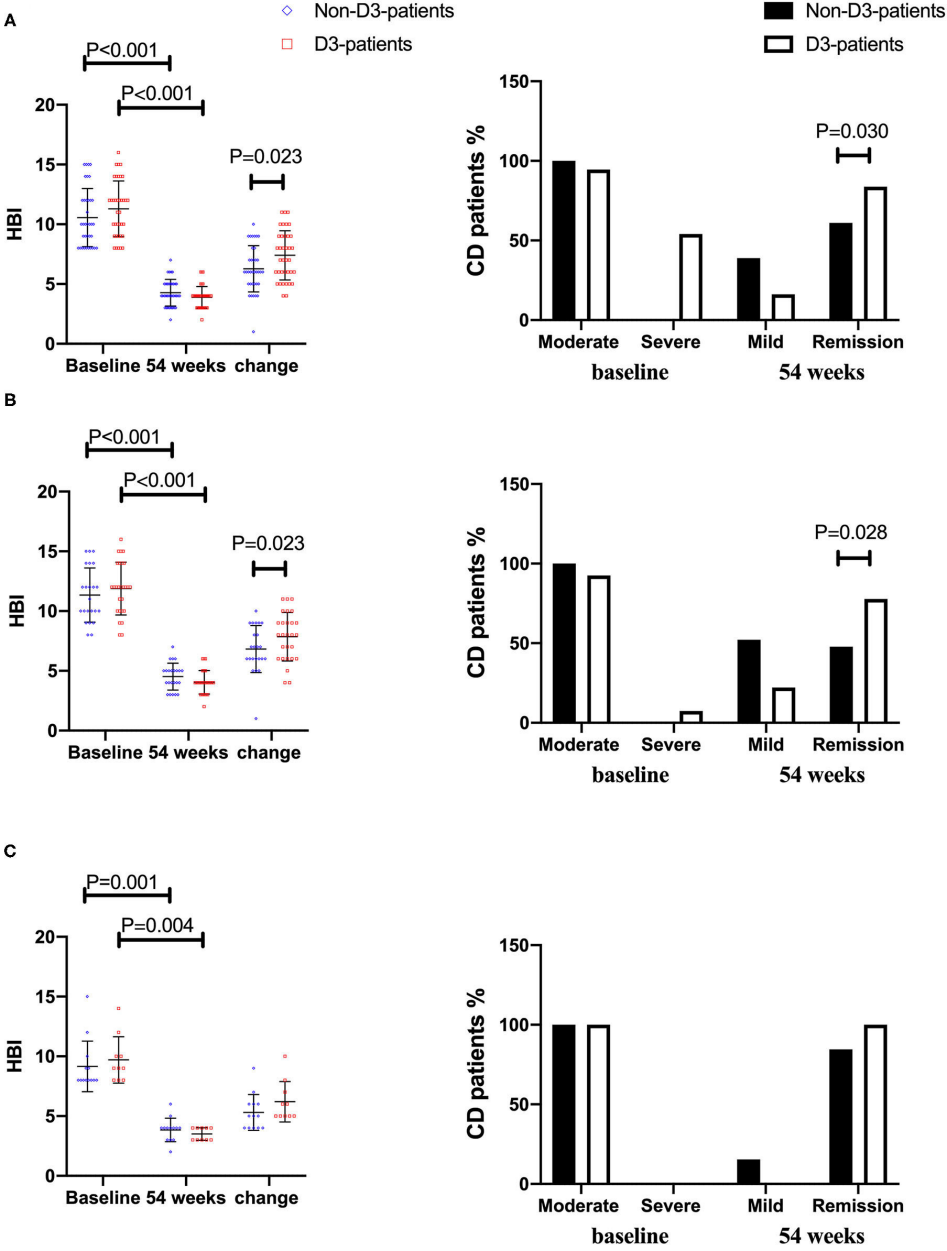
The changes of disease activity between baseline and week 54 in CD patients receiving IFX therapy. **(A)** In total cohort. **(B)** In vitD deficient group. **(C)** In vitD non-deficient group.

On the follow-up visit for 54 weeks, 72.6% (53/73) patients receiving IFX treatment achieved clinical remission, and 27.4% (20/73) were degraded into mild activity. The overall median HBI was 10 at baseline, and decreased to 4 at week 54 (*P* < 0.001, paired Wilcoxon's signed rank test). However, the increase of remission rate in D3-patients was more significant than in non-D3-patients at week 54 (83.8 vs. 61.6%, *P* = 0.030, chi-square test). The similar trend was obtained for the decrease of HBI between baseline and week 54 in D3-patients when compared with non-D3-patients (7.41 ± 3.0 vs. 6.28 ± 2.75, *P* = 0.023, student's *t*-test) (Figure 4A). In addition, the average/median levels of BMI, Hb and Alb significantly increased, while CRP and ESR significantly decreased in either patients with or without oral vitD3 supplementation after 54-week IFX treatment (week 54 vs. baseline, all *P* < 0.05, paired student's *t*-test or paired Wilcoxon's signed rank test). However, the differences in changes of these parameters did not reach statistical significance between D3-patients and non-D3-patients at week 54 (all *P* > 0.05, student's *t*-test or Wilcoxon's signed rank test) (Figures 2A–F).

After adjustment of age, gender, smoking status, disease extent, disease behavior, perianal lesions, upper GI involvement, prior surgical history, concomitant use of AZA, the multivariable logistic regression analysis was further conducted to determine the association of vitD3 supplementation with the achievement of clinical remission in the total cohort. The results suggested that vitD3 supplementation was an independent factor for improving the remission rate of IFX-treated CD patients (β = −1.667, *P* = 0.015) (Table 3).

**Table 3:** The influence of vitamin D3 supplementation on the clinical remission in CD patients treated with IFX (logic regression, enter) (*n* = 73).

**Covariants**	***B***	**Standard error**	***P***	***OR***	**95%*CI***
Sex	−0.394	0.706	0.576	0.674	0.169–2.688
Age^*^	−0.067	0.848	0.937	0.936	0.178–4.928
Smoking status	−1.191	1.421	0.402	0.304	0.019–4.924
Prior surgical history	−0.372	0.753	0.622	0.690	0.157–3.019
Disease location	0.224	0.350	0.523	1.251	0.630–2.494
Disease behavior	0.442	0.343	0.198	1.556	0.794–3.050
Perianal lesions	−0.494	0.781	0.527	0.610	0.132–2.821
Upper GI involvement	1.641	1.345	0.222	5.159	0.370–71.906
Concomitant use of AZA	−0.510	0.792	0.520	0.601	0.127–2.835
Supplementation or not	−1.667	0.683	**0.015**	0.189	0.050–0.719

* *Age was transformed as categorical variables where age <40 year-old = 0, age ≥ 40 years old = 1.*

*GI, gastrointestinal; AZA, azathioprine*.

In D3-patients, the multivariable linear regression model was established to explore the influence factors for the beneficial role of vitD3 supplementation. The following variables were included: age, gender, smoking status, disease extent, disease behavior, perianal lesions, upper GI involvement, prior surgical history, concomitant use of AZA, BMI, Hb, PLT, CRP, ESR, Alb, baseline 25(OH)D, and HBI. As a result, the concomitant use of AZA and baseline HBI were shown to be independently and positively correlated with the decrease of HBI in those CD patients (*B* = 0.645, *P* = 0.028; *B* = 1.090, *P* < 0.001, respectively) (Table 4).

**Table 4:** Influence factors on the decrease of HBI in CD patients supplemented by vitamin D3 (liner regression, enter) (*n* = 37).

**Covariants**	***B***	**Standard error**	***t***	***P***	**95%*CI* for B**
Age	−0.005	0.014	−0.342	0.736	−0.034–0.024
Sex	0.764	0.542	1.411	0.174	−0.369–1.898
Smoking status^§^	−0.867	1.142	−0.759	0.457	−3.258–1.523
Perianal lesions^§^	0.059	0.304	0.193	0.849	−0.578–0.695
Upper GI involvement^§^	0.731	0.607	1.204	0.243	−0.540–2.002
Concomitant use of AZA^§^	0.645	0.272	2.373	**0.028**	0.076–1.213
Prior surgical history^§^	−0.064	0.425	−0.150	0.882	−0.954–0.826
Disease location^§^	0.177	0.178	0.992	0.334	−0.196–0.549
Disease behavior^§^	−0.152	0.192	−0.794	0.437	−0.554–0.249
BMI	−0.038	0.085	−0.450	0.658	−0.216–0.140
Hb	0.005	0.016	0.283	0.780	−0.030–0.039
PLT	−0.005	0.003	−1.751	0.096	−0.011–0.001
CRP	0.000	0.010	−0.031	0.976	−0.022–0.021
ESR	−0.021	0.013	−1.588	0.129	−0.049–0.007
Albumin	0.013	0.041	0.313	0.758	−0.074–0.100
Baseline levels of Vitamin D	−0.009	0.028	−0.301	0.767	−0.068–0.051
Baseline HBI	1.090	0.085	12.860	** <0.001**	0.913–1.268

§ *Transformed as dummy variable.*

*BMI, body mass index; Hb, hemoglobin; PLT, platelet; CRP, C-reactive protein; ESR, erythrocyte sedimentation rate*.

### Influence of VitD3 Supplementation on the Disease Activity Stratified by Baseline VitD Status

To clarify whether vitD status at baseline influenced the beneficial effect of vitD3 supplementation in CD patients, the change of disease activity and relevant biochemical parameters were separately analyzed in the subgroups of vitD deficient (<20 ng/mL, *n* = 50) and non-deficient (≥20 ng/mL, *n* = 23). In each subgroup, CD patients were further divided into supplementary (D3-patients) and non-supplementary group (non-D3-patients). Overall, D3-patients and non-D3-patients were well-balanced with respect to demographic and disease characteristics in either vitD deficient or non-deficient group, respectively (all *P* > 0.05, student's *t*-test, Wilcoxon's signed rank test or chi-square test) (Table 1, Figures 2G–R). The exception is the gender distribution in non-deficient group, which presented a higher ratio of male in sub-non-D3-patients than in sub-D3-patients (*P* = 0.007, chi-square test) (Table 1).

When comes to vitD deficient subgroup, after 54-week of vitD3 supplementation, D3-patients manifested a greater increase of 25(OH)D than non-D3-patients did (1.65 ± 5.64 vs. 7.19 ± 6.25, *P* = 0.002, student's *t*-test) (Figure 3C). Moreover, the remission rate, together with the decrease of HBI, in D3-patients was higher than that in non-D3-patients (*P* = 0.028, chi-square test) (Figure 4B). The multivariable logistic regression model showed that vitD3 supplementation was an independent factor for improving the remission rate of patients under IFX therapy (*B* = −1.919, *P* = 0.019), after adjustment of age, gender, smoking status, disease extent, disease behavior, perianal lesions, upper GI involvement, prior surgery, concomitant use of AZA (Table 5). However, this significant difference of changes in 25(OH)D concentration and remission rate between D3-patients and non-D3-patients did not appear in non-deficient group (all *P* > 0.05, paired student's *t*-test or chi-square test) (Figures 3D, 4C).

**Table 5:** Influence of vitamin D3 supplementation on the clinical remission in CD patients with vitamin D deficiency (logic regression, enter) (*n* = 50).

**Covariates**	***B***	**Standard error**	***P***	***OR***	**95%*CI***
Sex	0.833	0.894	0.351	2.301	0.399–13.257
Age^*^	1.127	1.213	0.353	3.085	0.286–33.277
Smoking status	−2.473	1.785	0.166	0.084	0.003–2.788
Prior surgical history	−0.094	1.150	0.935	0.911	0.095–8.681
Disease location	0.532	0.459	0.247	1.702	0.692–4.186
Disease behavior	0.749	0.428	0.080	2.115	0.914–4.895
Perianal lesions	−1.093	0.966	0.258	0.335	0.050–2.228
Upper GI involvement	1.581	1.492	0.289	4.860	0.261–90.496
Concomitant use of AZA	−0.539	0.925	0.560	0.583	0.095–3.577
Baseline disease severity^#^	1.754	2.028	0.387	5.776	0.109–307.448
Supplementary or not	−1.919	0.820	**0.019**	0.147	0.029–0.732

* *Age was transformed as categorical variables, that was age <40 years old = 0, age ≥ 40 years old = 1.*

# *Disease severity was defined by HBI, and transformed as categorical variables, that was moderate activity = 0, severe activity = 1*.

### Effects of VitD3 Supplementation on Metabolism of Calcium and Phosphorus

**Figure 5:**
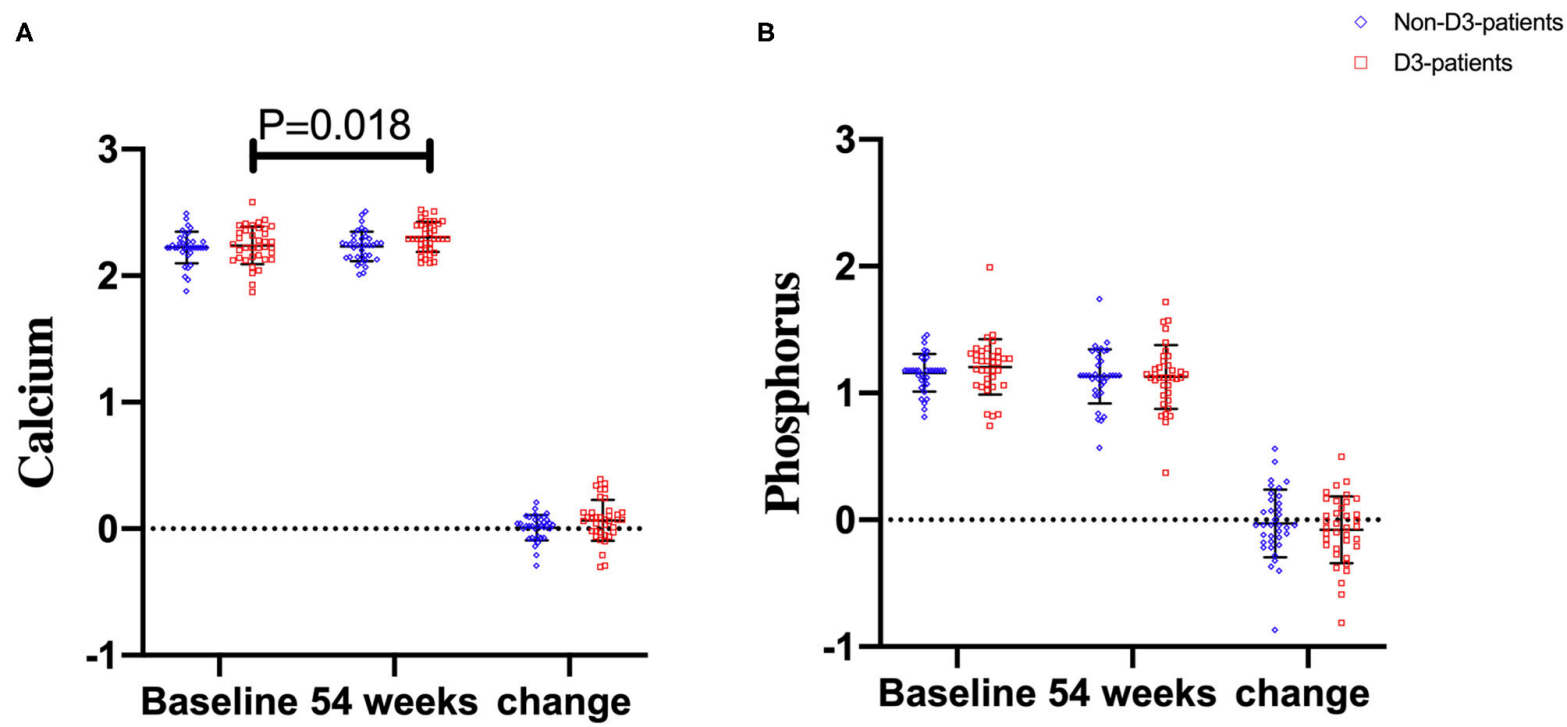
The changes of calcium and phosphorus levels between baseline and week 54 in CD patients receiving IFX therapy. **(A)** Calcium levels. **(B)** Phosphorus levels.

Serum calcium and phosphorus levels at baseline and week 54 were also examined. No statistical differences in the two parameters at baseline were observed between D3-patients and non-D3-patients (*P* = 0.617, 0.172, respectively). At week 54, changes of serum calcium or phosphorus also did not reach statistical significance between D3-patients and non-D3-patients (*P* = 0.074, *P* = 0.567, respectively) (Figures 5A,B). Despite the mean concentration of calcium in D3-patients significantly raised at week 54 (*P* = 0.018, paired *t*-test), all data did not exceed the upper limit of normal calcium level (Figure 5A).

### Influence of VitD3 Supplementation on the Expression Profiles of Th-Cell-Related Cytokines in CD Patients

**Figure 6:**
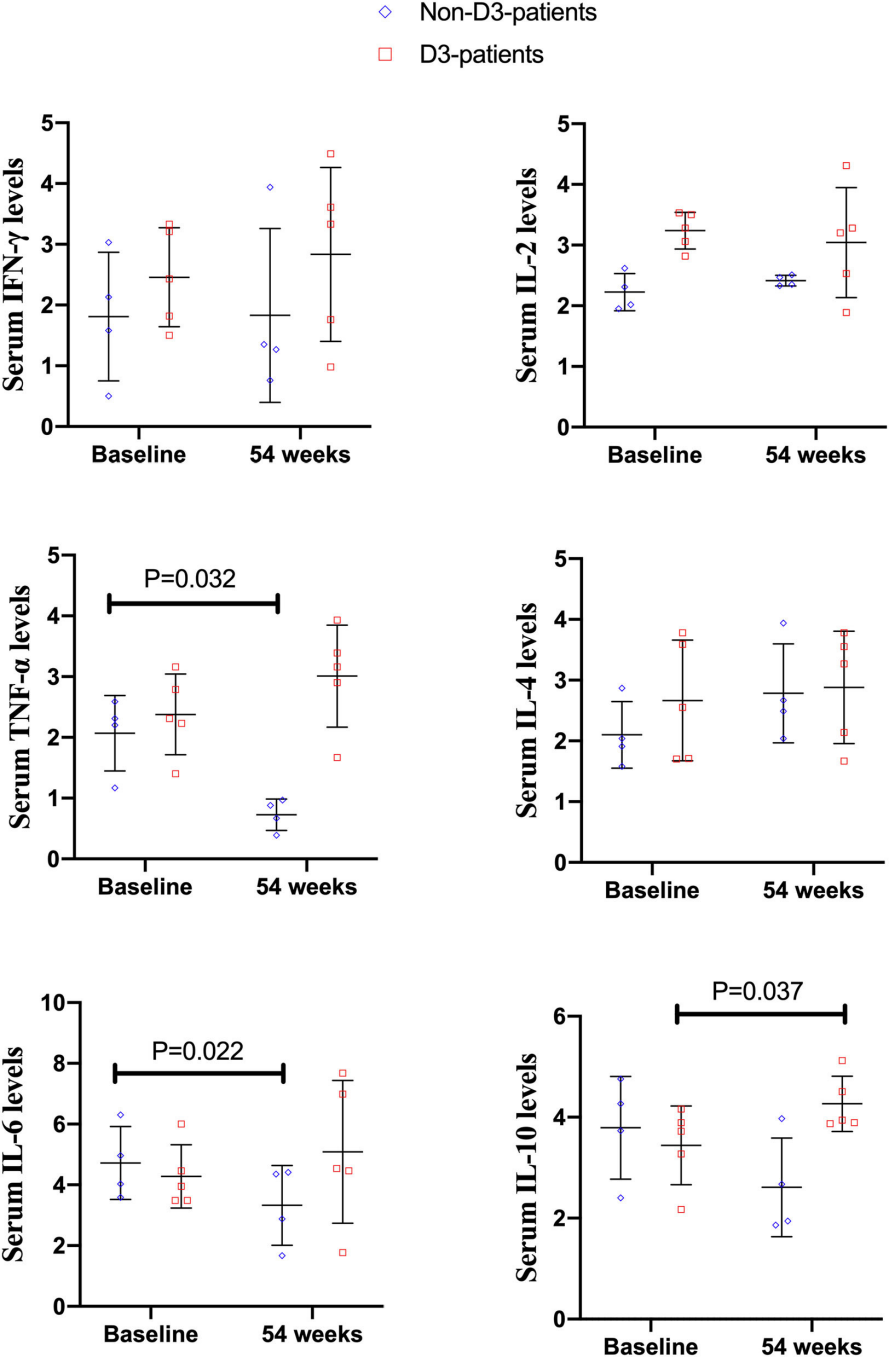
The changes of cytokines levels between baseline and 54 weeks in CD patients receiving IFX therapy.

Among 73 enrolled patients, nine patients (four patients without vitD3 supplementation, five patients supplemented by vitD3) had blood sample at baseline and week 54. Serum TNF-α and IL-6 level in non-D3-patients significantly reduced at 54-week compared to their baseline values (*P* = 0.032, 0.022, respectively). There were no significant differences of IL-2, IL-4, IL-10, and IFN-γ between baseline and week 54 (all *P* > 0.05). However, the cytokine profiles in D3-patients were quite distinct from non-D3-patients. Serum level of IL-10 at week 54 had a remarkable increase in D3-patients (*P* = 0.037). No statistically significant change was found in other cytokines (Figure 6).

## Discussion

The role of vitD in immune-related diseases has been investigated extensively for over a decade. There is ample evidence that oral vitD3 supplementation potentially plays a beneficial role in reducing activities of these diseases, including CD (12, 24). Yang et al. previously revealed that the 24-week supplementation with up to 5,000 IU/d vitD3 could effectively reduce the CD activity index (CDAI) scores in a total of 18 CD patients (25). However, no consistent conclusions could be drawn on the benefit of vitD3 supplementation for the outcome of CD from those similar clinical trials (24, 25). And the synergistic effect between vitD3 supplementation and IFX treatment in CD patients have not been highlighted. The present study mainly found that vitD3 supplementation was conducive to the improvement of IFX effectiveness in CD patients, especially for those with vitD deficiency or high disease activity.

Above all, we noted that the 125 IU/d vitD3 supplementation for 54 weeks could significantly raise serum 25(OH)D from 15.07 to 20.33 ng/mL in D3-patients. By contrast, serum 25(OH)D kept basically stable in non-D3-patients throughout the study. These observations suggested that additional oral vitD3 supplementation, rather than IFX alone, was responsible for the increase of serum 25(OH)D in CD patient under IFX treatment. Several double-blind randomized placebo-controlled studies (RCT) have attempted to investigate the influence of vitD3 supplementation on serum 25(OH)D in CD patients (26–29). The data from a cohort of Irish CD patients in remission showed that daily 2,000 IU vitD3 oral supplementation for 3 months was sufficient to raise 25(OH)D from 27.7 to 36.4 ng/ml in those patients (26). Jorgensen *et al*. reported that oral vitD3 treatment with 1,200 IU daily in Danish CD patients in remission could increase serum 25(OH)D from 27.6 to 38.4 ng/ml after 3 months, and the increased 25(OH)D was sustained throughout the 12-month vitD3 treatment period (27). In addition, de Bruyn et al. found that there was an elevation of the serum 25(OH)D from 16.8 to 32.0 ng/ml in CD patients who took oral 25,000 IU/week for 26 weeks (28). Intriguingly, the three studies exhibited that serum 25(OH)D decreased or stayed stable in the placebo group. However, a Canadian double-blind RCT demonstrated a significant increase of serum 25(OH)D (29.4–64.3 ng/mL) in CD patients who are in remission, after receiving 10,000 IU/d vitD3 for 12 months. By contrast, those patients receiving 1,000 IU/d had no significant change of 25(OH)D (28.5 to 33.1 ng/mL) (29). It is well-known that there is still no recommended dose and regimes for vitD3 supplementation in either CD or other immune-related diseases. Even for a healthy adult, the Institute of Medicine (IOM) recommends 600 IU/d (30), while the Endocrine Society recommends 1,500–2,000 IU/d (21). In a word, the above inconsistent observations make it imperative to explore the suitable dose, regimes and frequency of vitD3 supplementation in CD patients.

As well, there is a wide range of individual variation in response to the same dose vitD3 supplementation due to variances in initial levels and gastrointestinal absorption. Such a viewpoint was also validated by our study, which showed that upper GI involvement and baseline 25(OH)D were significantly related to the change of 25(OH)D in Chinese CD patients receiving vitD3 supplementation. More specifically, the patients with upper GI involvement or higher baseline 25(OH)D had a lower increase of serum 25(OH)D after vitD3 supplementation. On the one hand, vitamin D is absorbed by means of a simple passive diffusion process in the brush-border (apical) membrane of the enterocytes (31). The dysfunction or structural disruption of small intestine may interfere with vitamin D absorption. Leichtmann et al. compared the intestinal absorption of vitD3 in CD patients with different resections of the small bowel: smaller (<100 cm), intermediate (100–300 cm), and larger (>300 cm) resections (32). They found that intestinal absorption of vitD3 was reduced in such CD patients, with the degree of impairment increasing with greater resection (32). Therefore, we supposed that the negative impact of upper GI involvement on the increase of 25(OH)D levels probably due to their less intestinal absorption of vitD3. On the other hand, Lorentzon and Danielsson confirmed that the intestinal vitD absorption was in turn affected by individual vitD status (33). After administrating intragastrically [^3^H]-labeled vitD3 ([^3^H]vitD3) to rats and monitoring serum radioactivity for 3 days, they found that rats with vitD deficiency accumulated significantly higher levels of radioactivity, which implicated a higher intestinal absorption in states of vitD-deficiency.

Subsequently, we found that the increase of remission rate and the decrease range of HBI were more significant in D3-patients than in non-D3-patients after 54-week of IFX treatment. The logistic regression analysis further proved that vitD3 supplementation independently affected the increase of remission rate in this cohort of IFX-treated patients, suggesting that the concomitant vitD3 supplementation was beneficial for improving the effectiveness of IFX. As mentioned above, Zator et al. reported that IBD patients with insufficient vitD (<30 ng/mL) were prone to earlier cessation of anti–TNF-α therapy (HR, 2.13) (19). Likewise, Winter et al. found that IBD patients with normal vitD levels at the initiation of anti-TNF-α therapy had 2.64 folds of increase for remission at 3 months compared to patients with low vitD levels (34). In addition, we found that patients with a higher CD activity at baseline and concomitant use of AZA had a more significant decrease of HBI in the patients after 54-week of vitD3 supplementation. In clinical practice, the combination of IFX and AZA was commonly used in the treatment of CD patients with high disease activity and poor predicated prognosis. Colombel et al. also demonstrated that such a combination therapy had a greater efficacy than IFX or AZA monotherapy to mitigate the progression of CD (35). In this regard, the above findings of our study suggested that the beneficial effect of vitD3 supplementation was more evident in patients with higher disease activity.

Currently, there is an ongoing debate about the minimal or optimal vitamin D levels in patients with chronic inflammatory diseases, including IBD. The reference level of vitamin D is mainly based on its skeletal effect, whereas the effect of vitamin D on extra-skeletal functions remains uncertain. Serum levels of vitD higher than 30 ng/mL are speculated to be necessary for immunomodulatory and non-skeletal effect in some reports, however, some investigators have suggested vitD levels >40 ng/mL may be needed. In the present study, CD patients were further assigned into two subgroups according to serum 25(OH)D level: the deficient group (<20 ng/ml) and non-deficient group (≥20 ng/ml). Consequently, we found that the benefit of vitD supplementation was significant in patients with deficient vitD, rather than those with non-deficient vitD. This finding implied that additional supplementation of vitD3 on top of IFX therapy may be more beneficial for the patients with vitD deficiency.

The safety of oral vitD3 was also considered carefully in the present study. Theoretical risks for this supplementary therapy include hypercalcemia, hypoparathyroidism, and renal calculi. Previous studies have indicated that vitD3 supplementation, ranging from 1,200 to 10,000 IU/d, can be used safely (27, 29). Consistent with these observational studies, we did not observe any episode of hypercalcemia or hyperphosphatemia during 54 weeks of 125 IU/d vitD3 supplementation.

It has been suggested that cytokines secreted by CD4^+^ T cells are associated with the effectiveness of IFX in CD (10, 11). Ogawa et al. previously reported that higher levels of IL-17A, IL-23, and IL-12 at baseline could predict poor therapeutic response to maintenance IFX therapy in CD patients (10). To explore the potential mechanism of the oral vitD3 in improving IFX effectiveness, a subset of IFX-treated patients was selected to determine the serum levels of CD4^+^ T cells related cytokines (IL-2, IL-4, IL-6, IL-10, TNF-α, and IFN-γ) before and after vitD3 supplementation. The results showed that the cytokine profiles in D3-patients were quite distinct from those in non-D3-patients. Firstly, we found that the serum levels of IL-6 and TNF-α were decreased in the non-D3-patients following 54-week IFX administration. Theoretically, TNF-α and IL-6 are important pro-inflammatory cytokines and known to be widely involved in pathogenesis of CD. Elevated serologic and mucosal levels of these two cytokines have been found in patients with CD (36). In animal models of colitis as well as in small-scale therapeutic trials in CD, blocking IL-6 with monoclonal antibodies has been demonstrated to be effective (37). Moreover, IBD patients with a reduction in IL-6 from baseline to 10 weeks receiving biologic therapy have shown to be a 4.7-fold higher probability of achieving a clinical response at 12 months compared to those without IL-6 reduction (38). Summarizing these findings, we speculated that IL-6 and TNF-α may be the potential targets related to the therapeutic effects of IFX in CD, though the mechanism was not fully understood. Consistent with our study, Engstrom et al. noticed that IL-6 and TNF-α levels were lower in IBD patients after 12 weeks IFX administration (39). Kato et al. noted that IL-6 and TNF-α levels were reduced in CD patients after the initial IFX infusion (2 weeks), but not in 14 weeks (40). Mizutani et al. found that the serum levels of IL-6, rather than TNF-α, were decreased in CD patients after 4-week IFX administration (41). Therefore, further studies are needed to clarify the exact association among IFX therapy, drug effectiveness and changes of IL-6 and TNF-α, and various confounding factors, such as study samples, treatment time, and measurement method should be taken into consideration. Secondly, we found that only IL-10 levels were increased in D3-patients after additionally oral VitD3 supplementation. Most of the available evidence has suggested that IL-10 signaling was closely involved in anti-TNF-α therapy. A typical example is that the colitis developed in IL-10 mutant mice are refractory to anti-TNF-α therapy (42). Koelink et al. confirmed that the efficacy of anti-TNF-α agents in resolving intestinal inflammation is critically dependent on IL-10 signaling in macrophages (43). As for the immunoregulatory function of vitD, vitD3 can facilitate the production of IL-10 and enhance IL-10-mediated immunomodulatory function. Moreover, vitD signaling could shift T-cell responses away from pro-inflammatory Th1/Th17 toward anti-inflammatory Treg response and induce the proliferation and differentiation of Treg, which was an important source of IL-10 (44). Therefore, we speculated that the beneficial role of vitD3 supplementation in IFX-treated CD patients might partially arise from the induction of IL-10.

Of course, the present study has several limitations. Firstly, it was a single-center study with a small sample size, especially for the analysis of cytokine profiles. A larger multi-center study would provide a more robust and representative study data. Secondly, many patients were excluded because their medical data were incomplete. A prospective cohort trial monitoring vitamin D levels after initiating IFX therapy would enable us to better elaborate the relationship between vitD3 supplementation and IFX response in CD patients. Thirdly, this study only included 3 older patients (4.1%). Unlike young individuals, the elderly was more susceptible to vitD deficiency and bone diseases such as osteoporosis. More evidence was needed to further clarify this beneficial effect of vitD3 supplementation in older patients with CD. Fourth, the response to IFX was only determined at week 54 after initiation of vitD3 supplementation in our study. A longitudinal study with longer follow-up, evaluating dynamic change of 25(OH)D levels as well as association with other IBD related health outcomes, should be considered and studied. Fifth, we did not collect data to rule out other possible confounders, such as dietary vitamin D intake and sunlight exposure. Finally, this study did not record the clinical symptoms related to vitamin D intoxication, such as irritability, anorexia, nausea, vomiting, and diarrhea. Further prospective cohort studies would be considered.

## Conclusions

Daily supplementation of vitD3 (125 IU/d) is a safe and effective treatment to raise the 25(OH)D level in CD patients under IFX treatment. Moreover, this mode of supplementation could improve the clinical response to IFX, especially for those with vitamin D deficiency and high disease activity, probably via up-regulating IL-10 expression. Although the present study showed that this dose of vitD3 supplementation could effectively increase 25(OH)D level without any signs of hypervitaminosis D, we could not demonstrate the general applicability of this supplementation mode in all CD patients. Therefore, a longitudinal study is needed to investigate the association of various dose and regimes of vitD3 supplementation with the relevant clinical outcomes of CD.

## Data Availability Statement

The raw data supporting the conclusions of this article will be made available by the authors, without undue reservation.

## Ethics Statement

The studies involving human participants were reviewed and approved by the Ethics Committee of The Second Affiliated Hospital of Wenzhou Medical University. The patients/participants provided their written informed consent to participate in this study.

## Author Contributions

S-lX and YJ: study concept and design. Q-jM, X-xS, D-pL, and G-lM: acquisition of data. S-lX, HW, and S-gC: analysis and interpretation of data. S-lX: drafting of the manuscript. YJ: critical revision of the manuscript for important intellectual content. All authors reviewed the paper and approved the final version.

## Funding

The project was supported by grants from Zhejiang Provincial Natural Science Foundation (Grant number: LY17H030011), Chinese Medicine Research Program of Zhejiang Province (Grant number: 2019ZB075), Wenzhou Science and Technology Bureau Foundation (Grant number: Y2020011), and Lin He Academician Workstation Program (Grant numbers: 19331101 and 19331206).

## Conflict of Interest

The authors declare that the research was conducted in the absence of any commercial or financial relationships that could be construed as a potential conflict of interest.

## Publisher's Note

All claims expressed in this article are solely those of the authors and do not necessarily represent those of their affiliated organizations, or those of the publisher, the editors and the reviewers. Any product that may be evaluated in this article, or claim that may be made by its manufacturer, is not guaranteed or endorsed by the publisher.

## Abbreviations

25(OH)D: 25-hydroxyvitamin D
Alb: Albumin
AZA: Azathioprine
BMI: Body mass index
CBA: Cytometric Bead Array
CD: Crohn's disease
CDAI: Crohn's disease activity index
CRP: C-reactive protein
ESR: Erythrocyte sedimentation rate
Hb: Hemoglobin
HBI: Harvey Bradshaw Index
IBD: Inflammatory bowel diseases
IFX: Infliximab
IOM: Institute of Medicine
IQR: Inter-quartile range
PLT: Platelet
SD: Standard deviation
TNF: Tumor necrosis factor
Tregs: Regulatory T cells
UC: Ulcerative colitis
VDR: Vitamin D receptor gene.
